# Integrating AF4 and Py-GC-MS for Combined Size-Resolved Polymer-Compositional Analysis of Nanoplastics with Application to Wastewater

**DOI:** 10.1021/acs.analchem.5c01766

**Published:** 2025-07-11

**Authors:** Maria Hayder, Cloé Veclin, Aislinn Ahern, Aleksandra Chojnacka, Erwin Roex, Florian Meier, Gert-Jan M. Gruter, Annemarie P. van Wezel, Alina Astefanei

**Affiliations:** † Van’t Hoff Institute for Molecular Sciences, University of Amsterdam, Science Park 904, 1098XH Amsterdam, Netherlands; ‡ National Institute for Public Health and the Environment (RIVM), 3720BA, Bilthoven, Netherlands; § Postnova Analytics GmbH, Rankinestraße 1, 86899 Landsberg, Germany; ∥ Avantium Support BV, Zekeringstraat 29, 1014BV Amsterdam, Netherlands; ⊥ Institute for Biodiversity and Ecosystem Dynamics, University of Amsterdam, Science Park 904, 1098XH Amsterdam, Netherlands

## Abstract

Although nanoplastics are a widespread pollutant, their characterization and quantification in environmental samples remains challenging with no standard approach currently available. Here, we describe a novel workflow for nanoplastic analysis in environmental water samples, incorporating asymmetrical flow field-flow fractionation with multiangle light scattering (AF4-MALS) and pyrolysis-gas chromatography–mass spectrometry (Py-GC-MS) in an offline combination. The techniques complement each other as AF4-MALS enables sample cleanup and size separation down to about 1 nm, while Py-GC-MS identifies and quantifies polymers in each size fraction. Such a setup may provide comprehensive information about nanoplastic size distributions and polymer composition within a single workflow. After careful validation using standard polymer particles, we applied the method to wastewater samples. Our results show that the offline AF4-MALS-Py-GC-MS combination can identify certain nanoplastics in a complex environmental matrix. The mass quantification limits depend on the polymer type and range from 0.64 ng for PS to 180 ng for polyolefins. With our workflow, 8.8 ± 1.8 ng/mL polystyrene nanoplastics were quantified and polyvinyl chloride was potentially identified in untreated wastewater. Polyolefin and poly­(ethylene terephthalate) signals were below detection limits. While still in its early stages, this novel approach provides a promising foundation for particulate polymer analysis and highlights areas for further refinement, with the low recovery and potential of matrix interferences as drawbacks.

## Graphical abstract

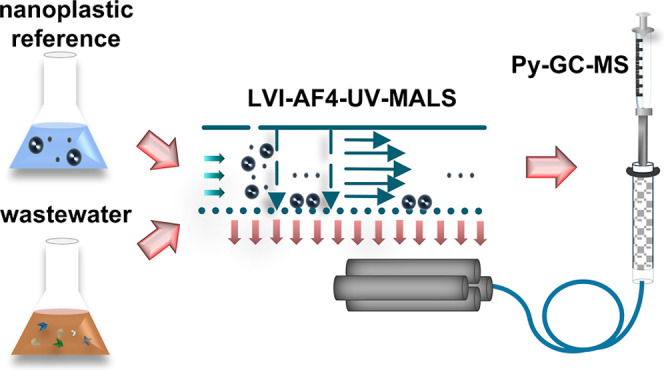

## Introduction

Plastic is an abundant environmental pollutant. Plastic waste undergoes degradation, forming micro- and nanoplastics (MNPs).^1,2^ While there is still no final consensus on the nanoplastics (NPs) definition, here we use this term for plastic particles sized 1–1000 nm.^3^ NPs differ from microplastics (1 μm-5 mm) in their transport properties and bioavailability.^4–6^

Detection, characterization, and quantification of NPs remain extremely challenging,^7^ with the following hurdles:

(i) Small size, which hampers detection by techniques conventional for microplastic research (e.g., FTIR or Raman spectroscopy do not have enough resolution^8^);
(ii) complex environmental matrices may cause interference or heteroagglomeration, which may lead to NP removal during analysis;
(iii) low mass concentrations, requiring instrumentation with low detection limits and high sensitivity.

Despite their expected ubiquity, research showing the actual presence of NPs in the environment remains limited^9–16^ with no routine method to characterize NPs in environmental samples.^17^ NP size distribution is a key in understanding the extent of pollution and its environmental impact.^18^ Asymmetrical-flow field-flow fractionation coupled to multiangle light scattering (AF4-MALS) has been applied to investigate NP presence in the environment.^9,19–22^ AF4 provides mild, nondestructive size-based separation, potentially useful for pretreating environmental samples.^15^

Particle size distributions must be combined with polymer type for identification, which AF4 cannot provide. A common approach for chemical analysis and mass quantification of MNPs is pyrolysis-gas chromatography–mass spectrometry (Py-GC-MS),^8,23–26^ providing polymer-related information by thermally degrading polymers into identifiable fragments. Combining the two techniques could result in comprehensive size and polymer information on NPs. Until now, scarce work has focused on practically combining AF4 and Py-GC-MS.^15,21,27^ One reason for this may be technical challenges when connecting both instruments, such as sample dilution during AF4 separation and the incompatibility of common AF4 eluents (nonvolatile salts and surfactants) with Py-GC-MS.

In this work, we present a novel approach for NP analysis in environmental water samples. We explore the capabilities and limitations of an offline workflow combining AF4-MALS (size-based separation and size distribution measurements and sample cleanup) and Py-GC-MS (polymer identification and quantification). To address low concentrations of NPs in environmental samples, we developed a large-volume injection (LVI) method for AF4, allowing for injection of 10 mL of sample. We believe this work is a significant step toward improving analytical approaches urgently needed in the NP field.

## Materials and Methods

The experiments were conducted in three stages. First, the AF4-MALS and Py-GC-MS methods and the sample handling steps were developed and validated. Next, the complete workflow was tested on standard polystyrene particle suspensions to demonstrate its performance. Finally, the setup was applied to wastewater to evaluate its effectiveness and identify pitfalls for NP analysis in a real complex matrix.

### Chemicals

Carboxyl-functionalized polystyrene particles (diameters 50 nm,PSC50, and 200 nm,PSC200) were purchased from Polysciences Inc. (Warrington, PA, USA). For method development, particle suspensions were diluted in the carrier liquid (see below) to obtain working suspensions (concentrations below). Other chemicals are listed in Section S1.

### Wastewater Samples

Influent samples were obtained from the Dutch National Water Quality Surveillance program. Sampling machines installed on each Sewage Treatment Plant (STP) in The Netherlands collect influent samples proportional to the total volume flowing through the STP in 24 h.^28^ A half-liter bottle is filled from this thoroughly mixed sample and transported to the laboratory in a cold chain complying with international ISO 5667–10 ^29^ and national NEN-6600–1 standards for wastewater sampling by contracted transporters. Around 200 mL was collected from 5 random STPs in February 2024 and pooled into one sample (approximately 1 L), stored at 4 °C until analysis.

### AF4-MALS Measurements

AF4-UV-MALS measurements were performed using an AF4-UV-MALS system (AF2000 MultiFlow FFF system, Postnova Analytics, Landsberg am Lech, Germany) with an SPD-20A UV/vis absorbance detector operated at 280 and 254 nm (PN3212; Shimadzu, Kyoto, Japan) and a 21-angle MALS detector (PN3621). Data were acquired by the AF2000 control software version 2.1.0.1 (Postnova Analytics). More details and the scheme of the applied system can be found in Section S2 and Figure S1. A 10 kDa PES membrane and 350 μm spacer were employed. 0.25 mM ammonium carbonate was selected as a carrier liquid compatible with Py-GC-MS (volatility and clean thermal decomposition). A stability study (Section S3) showed that it does not enhance agglomeration in the AF4 (comparison with other salts in Table S1).

For the small volume injection (SVI, 1 μL), a PSC stock suspension (323 μg/mL final concentration) in carrier liquid was used. Injection was performed for 3 min using the autosampler (injection flow *F*_inj_ = 0.2 mL/min). For LVI (10 mL), the stock suspension was diluted in carrier liquid to a final concentration of 32.3 ng/mL so that after the injection, the analyte mass (323 ng) is the same as in SVI. The LVI injection was performed manually for the time deemed optimal after the initial experiments (see the Results and Discussion section). Recovery calculation and the flow rate program are described in Section S4. The fraction collection started 1 min later than the elution. Eight fractions were collected for 7 min each. Experiments were performed in triplicate.

### Py-GC-MS Measurements

Py-GC-MS measurements were performed on a Shimadzu GCMS-QP2010 Plus system (Kyoto, Japan) with an Optic-4 programmed-temperature vaporization (PTV) injector as the pyrolysis chamber (ATAS GL, Veldhoven, The Netherlands) and a Focus XYZ autosampler (ATAS). More details regarding the PTV are listed in Section S5. Target polymers (polystyrene, PS, polyvinyl chloride, PVC, polyethylene terephthalate, PET, polyethylene, PE and polypropylene, PP) were chosen due to their large production volume and the resulting expected abundance in the environment.^30^ 55 μL of the liquid sample was injected at the inlet temperature of 50 °C. Injection repeatability and absence of memory effects were confirmed. Pyrolysis was performed at 550 °C (Table S2). The GC oven temperature was ramped from 50 to 320 °C (Table S3). Target pyrolysis products and the corresponding *m*/*z* and *t*_*r*_ values were selected (Table S6). The MS was operated (Table S4) in the scan mode (*m*/*z* 60to 300). To establish the indicative *m*/*z* for targeted polymers, no quantification was required. To obtain clear, high signals, manually cut minimal amounts of each solid polymer were placed in glass inserts for injection, and pyrolysis was performed at a column flow of 1.2 mL/min and split flow of 200 mL/min at 550 °C, followed by the GC–MS measurement as described above.

For liquid injections, targeted polymers were dissolved (dissolution conditions are in Table S5). Stock solutions were diluted in THF. Calibration curves were measured for each polymer individually (Figure S4). Limit of detection (LOD) and limit of quantification (LOQ) values were determined based on 26 THF blanks (methodology in Table S6).^25^For PET, there were no visible signals in blanks. LOD and LOQ were determined from the calibration curves as the lowest signal-yielding concentration and the lowest concentration in the linear response range, respectively.^26^

### Sample Handling

The schematic representation of the proposed workflow is depicted in Figure 1. Directly before analysis, samples were sonicated (A) for 10 min three times in a RK510H bath (frequency 35 kHz, nominal power 160 W, Bandelin, Berlin, Germany), with 10 min breaks in between. Subsequently, samples were filtered (B) over 1 μm PES syringe filters into a glass container. After AF4 measurements (C), fractions were collected into glass tubes (D), which were then capped with Miracloth (rayon), frozen, and freeze-dried (E; Heto PowerDry LL1500 Freeze-Dryer, Thermo Fisher Scientific, Waltham, MA, USA). Filtration and freeze-drying validation procedures are described in Section S6.

**Figure 1:**
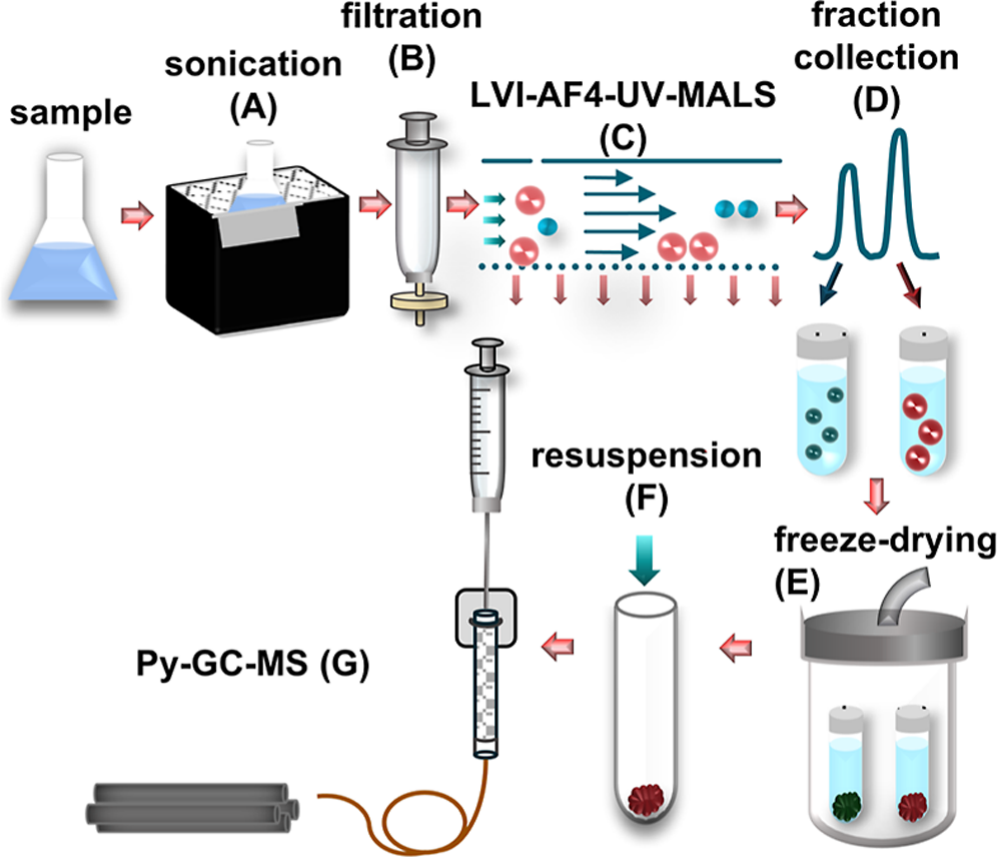
Schematic representation of the described workflow.

Next, samples were resuspended (F) in THF. One replicate was prepared by adding 500 μL of THF to each dry fraction and vortexing the tubes for 10 s. To increase the resuspension efficiency, the other two replicates were prepared by vortexing for 20 s and sonicating for 10 min (no great difference in signal intensities was observed in the results). THF was added slowly via the tube walls. Directly afterward, the suspension was transferred into glass vials. Such samples were injected into a Py-GC-MS system (G).

### Contamination Precautions and Quality Control

Cotton lab coats and nitrile gloves were worn. The work was performed in a fume hood: at least three blanks were run at each step of the workflow. Glassware was washed with ethanol and three times with ultrapure water before use. Utensils and vials were covered with aluminum foil or paper when not handled. Positive controls were included in the method validation.

For AF4 analysis, three SVI injections of the PSC50 stock suspension were performed after each membrane change to saturate the membrane.^31^ Between the LVI injections, the injection loop was flushed with three loop volumes of SDS-NaOH cleaning solution, four volumes of ultrapure water, and three volumes of the carrier liquid. In AF4, the PSC and wastewater injections were separated by two blanks (filtered carrier liquid) to prevent carryover.

In Py-GC-MS, a THF blank injection was performed between the samples. A quality control mixture (QCmix, a mixture of 3 ppm of each target polymer) was injected in triplicate in each batch as a positive control. To minimize the varying response of detector signals due to polymer interactions or the gradual decrease in instrument accuracy with each measurement (which could result in over- or underestimation of polymer masses), quantification was performed including the measurements of respective QCmix response for each batch. Each fraction was measured in duplicate as we already had triplicates of the fractions collected after the AF4-MALS measurements; this results in 6 replicates per fraction.

## Results and Discussion

### LVI-AF4-UV-MALS Method Development

Three injection times (55, 90, and 120 min) were investigated on PSC50 as smaller particles are more prone to agglomeration. At the injection flow rate used, the theoretical time needed to inject 10 mL is 50 min. To prevent sample residue losses in the loop, longer time is optimal, while prolonged injection and focusing increase the risk of undesirable particle–particle and particle–membrane interactions.^31^ The PSC50 recoveries in LVI-AF4 were 35 ± 4%, 46 ± 5%, and 43 ± 2%, respectively, at injection times of 55, 90, and 120 min, which is lower than that in SVI (65 ± 5%) and lower than typical values for conventional AF4.^32^ However, considering the challenges (long relaxation time required, particle propensity to agglomerate, and limited choice of carrier liquids), this recovery was deemed acceptable for the proof-of-concept study. The injection time of 90 min was selected.

The separation potential of the developed LVI-AF4 method was demonstrated by using a PSC mixture (Figure S2). The resolution between the two peaks was 1.95 ± 0.10. The elution times were 103.5 ± 0.4 min and 116.6 ± 0.6 min for PSC50 and PSC200, respectively. For LVI, the peaks eluted slightly earlier than that for SVI. The nearly absent void peak for LVI indicates proper focusing of most particles due to the long relaxation step (nearly no particles elute before relaxation is finished). For SVI, a void peak was observed, suggesting insufficient equilibration, likely caused by smaller particles (PSC50). Both SVI and LVI showed a residual peak after 40 min, when the crossflow was zero. This peak was higher for LVI compared to SVI. For LVI, peaks corresponding to PSC were smaller than that for SVI. This suggests that the lower recovery for LVI is partly caused by higher particle–membrane or particle–particle interactions, visible in the residual peak, though the direct measurement of the phenomena in the channel is technically challenging.

The radius of gyration (*r*_g_) values, as measured by the MALS detector (Sphere model, *r*_g_ calculation details in Section S2), align with those provided by the manufacturer. The *r*_g_ for PSC50 exhibited higher noise levels compared to PSC200, possibly resulting from the lower signal intensity related to the small particle size.

### Py-GC-MS Method Development

Pyrolysis products typical for the targeted polymers and their *m*/*z* values were found in the literature (Table S7) and confirmed by pyrolysis of solid samples in our instrument (Table S6).

The styrene trimer was chosen as the identifier for PS as the styrene monomer may be a product of other substances, and the styrene dimer was measured with a lower intensity than the trimer.

For PET, acetophenone was selected as the indicative pyrolysis product as it does not require derivatization, unlike benzoic acid. Although a derivatization agent might improve the measurement quality for the polar substances, its reaction mechanism is not yet fully understood. We thus avoided its use to minimize the risk of it reacting with other components of the complex samples.

Distinguishing between PE and PP is challenging as these two polyolefins have a similar structure and produce a similar mass chromatogram in the form of a “comb” with each peak corresponding to a different oligomer length. Additionally, they yield pyrolysis products sharing the same *m*/*z* values. Although the literature suggests *m*/*z* 69 and 97 can be used to distinguish between PP and PE (Table S7), we have observed both values in the separate analyses of individual polymers. A chromatogram of the PE and PP mixture proved that these two cannot be certainly separated (see Figure S3 and further discussion). We followed the literature in reporting *m*/*z* 69 as belonging to PP and *m*/*z* 97 to PE, however, only the polyolefin origin can be ascertained (see Section S7).

Conventionally, Py-GC-MS methods use solid standards, bringing large uncertainties to quantification.^23^ Here, we use liquid injection. Liquid injections of the same polymers were performed to confirm the presence of selected *m*/*z* and to observe the retention times (Table S6). Calibration curves were measured (Figure S4). We selected 55 μL of injection volume for Py-GC-MS, which is at the upper end of our instrumental possibilities without applying backflush. A detailed investigation on different mixtures and polymer ratios was out of the scope of this study; however, the presence of one polymer may influence the pyrolysis efficiency of another one.^33^ We ascertained the accuracy of our measurements by running QCmix in each batch instead.

### Sample Handling throughout the Workflow

Filtration recovery was 84 ± 7% and 105 ± 3% for PSC50 and PSC200, respectively. No fractogram deformations were observed after filtration. Calculated *r*_g_ did not show a significant difference between filtered and unfiltered samples (*p* = 0.91 and *p* = 0.49 for PSC50 and PSC200, respectively), proving that filtration does not affect the measurements. For blanks (carrier liquid), the changes in the UV peak area of the void and residual peaks before and after filtration were not statistically significant (*p* = 0.80 and *p* = 0.76, respectively, see Figure S5). However, they visibly differed from run to run, and thus, the contamination potential was further studied by Py-GC-MS. The results of Py-GC-MS revealed that the difference between filtered and unfiltered blanks was statistically significant only for PVC (*p* = 0.0002), where filtration increased PVC signal to a value balancing just below the LOD (21.1 ng). A possible explanation for this is that while we quantify PVC based on naphthalene intensity, this could also be a pyrolysis product of PES (the filter material), suggesting contamination with the filter residue during filtering. As the standard deviation of the PVC content in the filtered blanks is only 3.4%, we decided that blank correction is enough to prevent biasing results due to the filtration.

Freeze-drying recovery was 80 ± 1% and 83 ± 4% for PSC50 and PSC200, respectively. The recovery difference for the two particle sizes was not statistically significant (*p* = 0.37). The average recovery (82 ± 4%) was further used. Freeze-drying and resuspension did not increase the measured levels of any targeted polymer above LOD, except for PS, for which, however, the increase was not statistically significant (*p* = 0.2). The results presented in the further sections were blank-corrected based on the blank samples that had been subjected to the whole workflow. The theoretical total recovery of the sampling handling workflow obtained by multiplying the recoveries of respective stages is 73 ± 18% but only 32 ± 5% for PSC50 alone (Table 1). In the final experiments, an empirical recovery was used (see below).

**Table 1:** Summary of the Workflow Validation

stage of the workflow	negative controls	positive controls	validation
filtration	AF4 carrier liquid	PSC50, PSC200	R(PSC50) = 84 ± 7%; R(PSC200) = 105 ± 3%
			introduces statistically significant increase of PVC (as measured by Py-GC-MS)
LVI-AF4	filtered AF4 carrier liquid	PSC50, PSC200, and a 1:1 mixture of these	R = 94 ± 3% (PSC50 and PSC200 pooled)
			R = 46 ± 5% (PSC50 only)
freeze-drying	empty vials	PSC50, PSC200	R = 82 ± 4%
Py-GC-MS	THF	PS, PP, PE, PET, and PVC separately and in a mixture	
total	AF4 carrier liquid	1:1 mixture of PSC50 and PSC200	R_theoretical_ = 32 ± 5% (PSC50 only)
			R_theoretical_ = 73 ± 18% (PSC50 and PSC200 pooled)

### LVI-AF4-UV-MALS-Py-GC-MS Method Demonstration

A 1:1 mixture of PSC50 and PSC200 was separated and analyzed using the proposed workflow. The amounts of PS measured by Py-GC-MS were compared with those of the corresponding AF4 fractogram (Figure 2). The measured PS masses follow the PSC elution profile in AF4. The PS mass signal was higher in the later fraction of the PSC peaks, likely due to a slight time shift between the AF4 detection and fraction collection (the fraction collector is located after the AF4 detectors). The small increase in the PS signal in fractions 7 and 8 indicates that the residual peak contains some PSC, which may have agglomerated during the extended focusing step.^31^ Based on the calculated theoretical recovery (Table 1), the total mass of PS should be approximately 236 ng. However, the Py-GC-MS measurement only detected 23.2 ± 14.9 ng, which is much lower. We hypothesize that there are two potential causes for this discrepancy. First, resuspension may have been incomplete, resulting in some of the dried polymer material not being recovered for Py-GC-MS injection. Second, freeze-drying may alter the material’s morphology to much larger particles, subsequently prevented from being aspired during the Py-GC-MS injection. The resuspension efficiency was not directly measured due to practical constraints: the collected PS mass was too low for gravimetric analysis, and the geometry of the glass tubes prevented full-tube Py-GC-MS. Based on the known recovery in previous steps (73%) and the final Py-GC-MS signal (23.2 ng), we estimate a resuspension recovery of ∼10%. We postulate that resuspension and liquid injection in Py-GC-MS are the only way to quantitatively transfer the analytes between AF4 and Py-GC-MS. A future detailed investigation on different resuspension protocols could increase the recovery. The standard deviation was high for the fractions expected to contain most PS, possibly due to a larger influence on the measured mass in the case of loss of more/larger particles.

**Figure 2:**
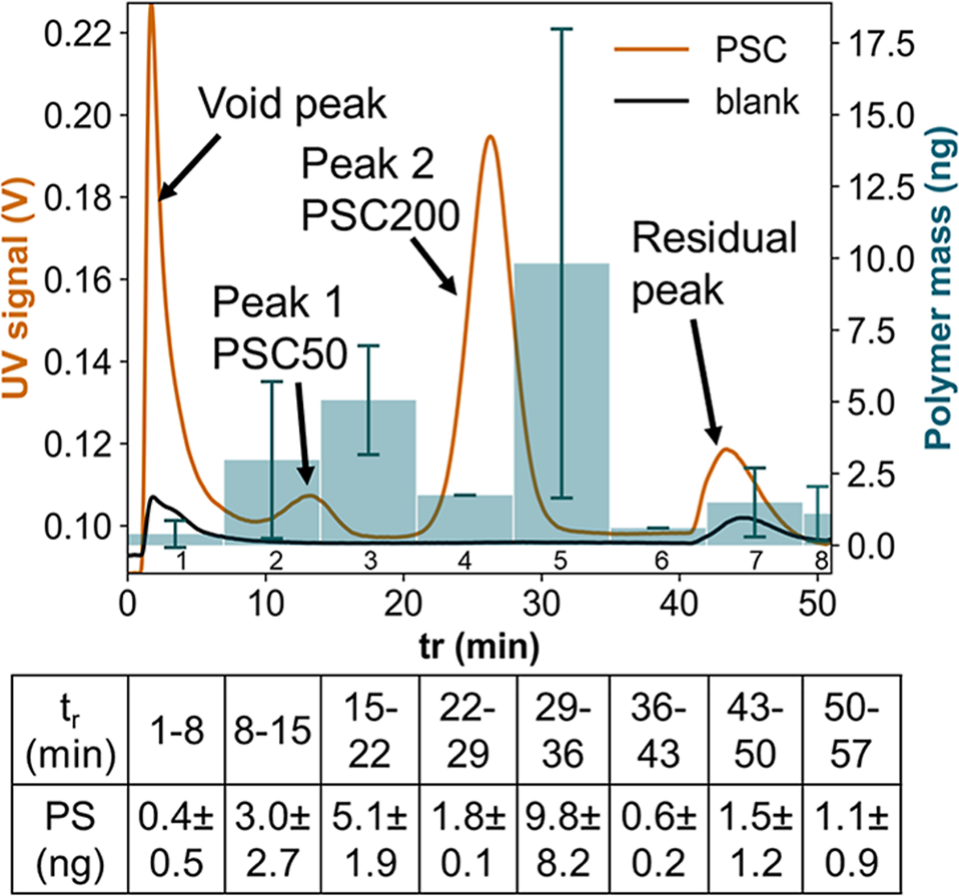
PSC analysis by the described workflow. UV fractograms: PSC standard mixture (orange trace) and blank (black trace). Blue bars: PS mass measured by Py-GC-MS.

This experiment demonstrates that our workflow can indeed detect polymer particles in the different size fractions collected by AF4, although quantification remains challenging due to the large losses during the resuspension step, leaving space for future optimization.

### Wastewater Analysis

The analysis results of an untreated wastewater sample processed using our workflow are shown in Figure 3. Figure 3A shows the overlay of the AF4-MALS fractograms (three LVI injections, conditions identical with those for PSC standards), where signals are high but irregular, displaying a broad tailing peak approaching baseline in the second half of the elution window and a residual peak. This suggests a continuous particle size distribution in the sample, as expected. Although the peak shapes are repeatable, size measurements by MALS vary between injections. This variability is probably related to the sample’s nature. Despite all efforts to homogenize the influent sample differences between replicates may still occur, e.g., in terms of particulate matter. The measured radii differ from the PSC elution profile (Figure S2) ranging from ∼270 to 700 nm, and vary more than those in the PSC mixture, possibly due to particle agglomeration in the organic matrix^34^ or different shapes of environmentally occurring NPs. In wastewater, NPs compose only a small fraction compared to other particulates and organic matter, which might influence MALS results, with larger particles masking the smaller ones. For radius calculations based on the MALS measurements, the errors generally increase with increasing analyte size.^35^ The size distribution pattern measured by MALS is repeatable. The particle sizes are highest in the first part of the fractogram, they later decrease and subsequently increase again, which may indicate mixed normal and steric elution, where the elution order is reversed,^36^ which complicates size separation and data evaluation. Particle agglomeration could contribute to such a behavior. Agglomeration depends on the matrix properties; thus, mixed-mode/steric elution is probably sample-dependent.

**Figure 3:**
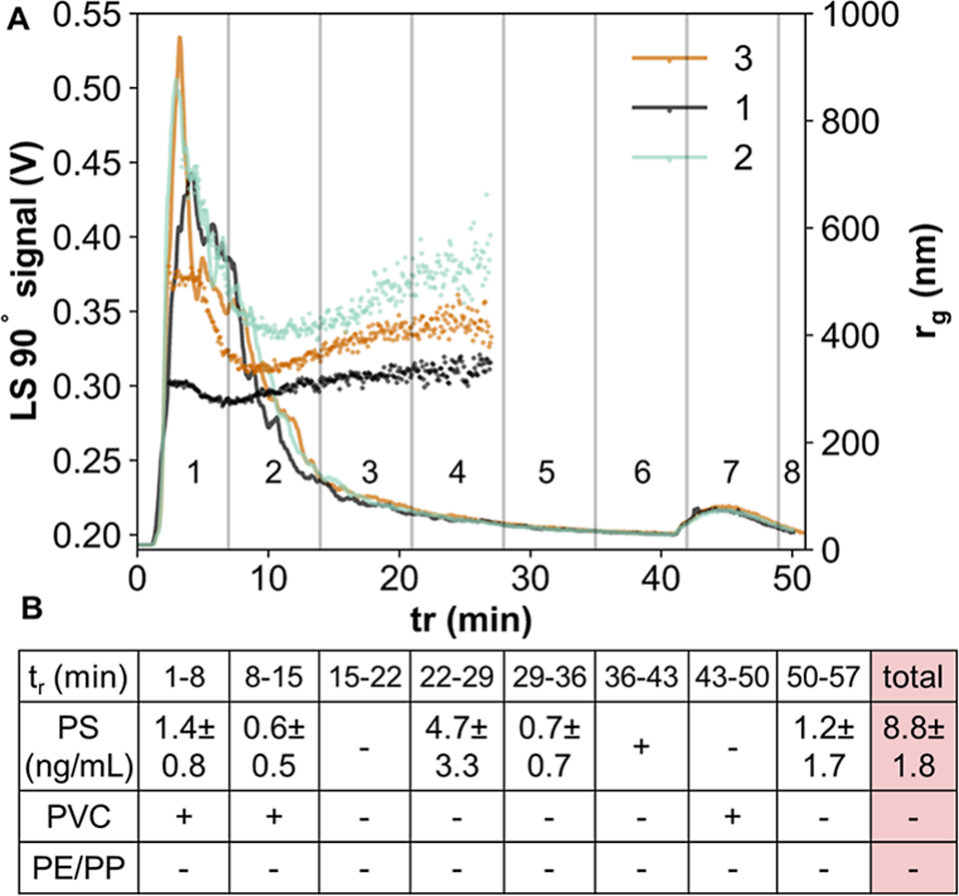
(A) AF4-MALS fractograms of three wastewater replicates; (B) polymer composition and concentrations detected in the untreated wastewater by Py-GC-MS in the respective fractions. “–” indicates signal < LOD, “+”-signal < LOQ.

The polymer composition and concentrations measured in the respective fractions by Py-GC-MS (blank-corrected) are shown in Figure 3B. PET was not detected in any fraction (LOD of 27.5 ng). This is potentially due to PET MPs occurring mostly as fibers,^37^ which may be retained on the filter at the beginning of the procedure. Future studies could consider the monomer quantification approach based on depolymerization.^38^ Polyolefins were below LODs (*m*/*z* 69:38.8 ng, *m*/*z* 97:86.0 ng) in all fractions, despite higher signal intensities than that in the blanks.

PVC was detected in fractions 1, 2, and 7, the latter corresponding to the residual peak, with measured levels between LOD (21.1 ng) and LOQ (54.0 ng). Its detection early in the fractogram may suggest that (i) PVC NPs at the low nanometer range are present in the wastewater (lower t_r_) and some of them interact with the membrane or aggregate forming the residual peak; and/or (ii) PVC NPs are present in the wastewater at the larger nanometer range and elute in the steric mode at the beginning of the fractogram, which would be well correlated with the *r*_g_ obtained; and/or (iii) a compound other than PVC, yielding naphthalene during pyrolysis, is present in the wastewater in the corresponding size. In complex samples, confusion of plastic signal with natural matter can hardly be excluded, which is an additional challenge in NP analysis. Presence of PVC NPs here is probable as PVC microplastics in wastewater have been reported.^39–41^ PS was quantified in fractions 1, 2, 4, 5, and 8. One of the three AF4 injections was excluded due to unrealistic variability in measured PS mass, leaving 4 Py GC MS measurements per fraction included in the calculations. Standard deviations are high. This might be a result of (i) low quantities of PS in the samples, making the quantification more challenging; (ii) matrix interferences, hampering analysis. The total PS mass measured across the fractions sums up to 6.4 ng, which, including the empirical recovery, points at 8.8 ± 1.8 ng/mL PS NPs in the untreated wastewater. Since the PS trimer is recommended as a unique PS indicator^23^ and the signals did reach the LOQ (0.64 ng), this confirms the ability of our workflow to analyze NPs in aqueous samples.

The LODs and LOQs differ between the polymers. The PS quantification and PVC detection alone does not exclude the presence of other polymers, which have higher LODs (from 27.5 to 86.0 ng, vs 0.22–21.1 ng for PS and PVC). Contrarily, literature suggests polyolefins as common MNPs in the aquatic environment,^24,40,42^ though the polymer diversity increases as particle size decreases.^40^ Given the high uncertainties in quantification, we do not aim at risk assessment at this stage but rather demonstrate the possibilities and limitations of our workflow.

### Relevance and Limitations of the Study

#### Overall Quality Control

We applied the quality assessment framework for microplastic analysis in freshwater.^42^ The experiments described here score 10/12 with three criteria deemed not applicable for a proof-of-concept study (“NA”) and excluded (Table 2). This is higher than the average score for wastewater studies reported at the time of publication (7.3/18 for all criteria).^42^ We argue that some of these guidelines should be adjusted for NP analysis. For future studies, we suggest relevant modifications to Table 2.

**Table 2:** QA/QC Assessment of the Study

criterion	definition by Koelmans et al.^42^	score	comments	suggestions for future nanoplastics research
sampling methods	location, treatment, date, sampling method, and materials used are reported	NA	exemplary sample for proof-of-concept	as for microplastics
sample size	at least 1 L	NA	exemplary sample of 1 L, of which 30 mL was analyzed for proof-of-concept; larger volumes might decrease standard deviations but further increase of injected volume in AF4 would be challenging as higher injection volume requires longer focusing time which decreases the recovery by enhancing particle–membrane interactions	required volumes were justified by the microplastics’ low number concentrations in water.^42^ NPs are smaller than microplastics, the sample is thus expected to be more homogeneous (though more prone to aggregation), thus lower volume might be acceptable
	can be smaller if the targeted mps sizes are smaller			we do not state a numerical value of the required volume. It should be chosen so as to decrease the variability of results and as feasible for the instrumentation
sample processing	storage/transport conditions, precleaned containers	1		as for microplastics
laboratory preparation	cotton lab coats, rinsed equipment	2		as for microplastics
clean air	laminar flow cabinet	1	fume hood with blanks run in parallel	as for microplastics
negative controls	triplicate blanks	2	elements of the AF4 system are made of (often less common) polymers. The Py-GC-MS system is made of metal (the lower risk of contamination)	further research needed to check if signals in negative controls can be linked to a specific step of the protocol
positive controls	protocol tested on known particles	2	there are fewer easily accessible types of standards for NPs than that for microplastics	positive controls should be as environmentally relevant (polymer types, shapes, and surfaces) as practically possible (lack of reference materials)
sample treatment	digestion of sample	NA	we focused on minimizing sample preparation, for simplicity and to reduce inherent measurement variability; AF4 has precleaning potential, which could be enhanced by using higher cutoff membranes, e.g., 100 kDa	with no standardized analysis protocol for NPs, no particular sample treatment is recommended but the treatment protocol must be either validated on relevant positive controls or reported elsewhere in literature specifically for NPs. Treatment influence on particle characteristics should be reported
analysis	polymer identification	2		no particular technique is recommended but polymeric nature must be confirmed. The analytical window should be clearly reported, all features specific for the instrumentation used should be considered, including possibility of biasing results
				this category could be split in two: (1) size analysis and (2) chemical analysis

### Alternative Strategies for Coupling AF4 to Py-GC-MS

Technical aspects of the AF4-Py-GC-MS combination remain challenging. This study outlines solutions that can be further optimized. First, fraction volumes of the AF4 eluent could be fine-tuned per sample. Provided that size fractions remain separated and that enough material is collected to reach the Py-GC-MS LODs, various fractionation strategies are possible. If certain regions of the fractogram are of particular interest, targeted collection could be performed.^43^

The most challenging part of this workflow is interfacing AF4 with Py-GC-MS. Due to the incompatibility between the aqueous AF4 carrier liquid and our Py-GC-MS method, which does not allow water, we used freeze-drying for water removal, enabling the transfer. While microplastic analysis by Py-GC-MS is often done by placing a solid sample directly in the pyrolysis cup, bypassing resuspension, we found this approach impractical here due to the minute amounts of dried material, which are difficult to transfer quantitatively. Instead, in our workflow, we resuspend dried samples in THF, assuming sufficient dispersion of the dried material for aspiration by the injector syringe. For a perfectly homogeneous Py-GC-MS sample, complete dissolution would be required. However, dissolving PE, PP, and PET requires toxic solvents, elevated temperatures, and time, lowering the feasibility for several fractions, especially when many samples are analyzed. This problem might be solved by further optimizing the resuspension procedure. However, for this, a set of NP reference materials of different polymers is indispensable, and such standards do not exist yet.

Liquid Py-GC-MS injections with an aqueous solvent are also possible after hardware modifications,^44,45^ instead which would eliminate the need for freeze-drying after AF4. We did not pursue this approach because AF4 introduces sample dilution.^31^ Given the low NP concentrations in environmental samples, we focused on fraction concentration rather than dilution. Alternatively, larger AF4 channel dimensions could accommodate larger sample volumes.

Finally, an online coupling is deemed desirable, leading to an automated platform, as described for SEC-Py-GC-MS,^44^ AF4-ESI-MS,^46^ AF4-ICP-MS,^47^ 2D-LC systems, etc. Beyond technical challenges exceeding those mentioned here, we claim that the dilution in the AF4 is a sufficient reason to postpone attempts at creating such a platform until a reliable and NP-selective preconcentration method is accessible.

### Limitations and Perspectives

Our workflow was validated using carboxylated PS spherical standards because of their sizes, commercial availability, and usability. However, such particles are not a realistic counterpart of environmental NPs,^48^ considering their regular shape, smooth surface, and well-defined density. Workflow performance was validated for these particles only. With current capabilities, no far-reaching conclusions should be drawn, especially regarding other polymers and irregularly shaped particles. When possible, experiments with different polymeric particles should be conducted to better understand the applicability of our approach.

Though LVI-AF4 enables overcoming low NPs’ concentrations in the environment, long injection times are troublesome. Upstream preconcentration could be considered, however, it would not decrease the overall workflow time nor labor, while it could decrease recovery of the smallest particles.^21^ LVI sample volume depends on the tailor-made loop and should be adapted to the sample. It should be large enough to overcome low analyte concentration but practical considering reasonable injection time and backpressure.

Wastewater is a complex matrix, bringing additional challenges besides those already defined for standard particles. Co-elution with natural organic matter interferes with the MALS and Py-GC-MS results. Sample digestion could be performed to alleviate this, although it encompasses additional risks (sample loss, particle degradation). Matrix complexity prevents reliable Py-GC-MS analysis of the unfractionated sample. The sample cleaning effect of LVI-AF4 should be studied on different sample matrices in the future.

The current sample losses limit the applicability of the workflow for NP quantification. The most urgent improvement needed is increasing the recovery, particularly in the resuspension step (e.g., by optimizing the suspending liquid composition or mechanical agitation) and, for smaller particles, during AF4 separation (by fine-tuning the AF4 method, e.g., the membrane or carrier liquid).

While focusing on adjusting the AF4 separation to the sample characteristics and interfacing it with Py-GC-MS, further work on the latter is advisable. On the future roadmap for methodological standardization, including more polymer types in the analysis (e.g., polyurethanes and nylons) and conducting studies on polymer–polymer and polymer–matrix interactions would enhance the potential of the proposed workflow and might lead to broader applicability and possible automation.

## Conclusions

A novel approach for NP analysis is proposed, aiming at simultaneous preconcentration, size estimation, and polymer identification/quantification for nanometer plastic particles. Technical capabilities, limitations, and possible solutions for the challenging LVI-AF4-MALSPy-GC-MS coupling are widely discussed for the first time. Our offline workflow was tested on standard particles and on wastewater, offering original insight into the potential for NP simultaneous size and mass analysis in complex matrices. PS NPs were successfully quantified in the wastewater. Recovery remains the greatest area for improvement, with an urgent need to optimize resuspension. Possible directions in platform development are suggested. Since AF4-Py-GC-MS is sought-after in the NP research, we believe our work could be used to broaden the knowledge on NP occurrence and fate.

## Supplementary Material



## Notes

Instrumental details, experimental parameters, LVI-AF4 carrier liquid optimization, fractograms, Py-GC-MS of target polymers (literature), polyolefin chromatograms, and calibration curves (PDF)

## References

[1] Pfohl P., Wagner M., Meyer L., Domercq P., Praetorius A., Hüffer T. (2022). Environmental Degradation of Microplastics:
How to Measure Fragmentation Rates to Secondary Micro- and Nanoplastic
Fragments and Dissociation into Dissolved Organics. Environ. Sci. Technol..

[2] Song Y. K., Hong S. H., Jang M., Han G. M., Jung S. W., Shim W. J. (2017). Combined Effects
of UV Exposure Duration and Mechanical
Abrasion on Microplastic Fragmentation by Polymer Type. Environ. Sci. Technol..

[3] Hartmann N. B., Hüffer T., Thompson R. C., Hassellöv M., Verschoor A., Daugaard A. E. (2019). Are We Speaking the
Same Language? Recommendations for a Definition and Categorization
Framework for Plastic Debris. Environ. Sci.
Technol..

[4] Gigault J., El Hadri H., Nguyen B., Grassl B., Rowenczyk L., Tufenkji N. (2021). Nanoplastics
are neither microplastics nor
engineered nanoparticles. Nat. Nanotechnol..

[5] Mitrano D. M., Wick P., Nowack B. (2021). Placing nanoplastics
in the context
of global plastic pollution. Nat. Nanotechnol..

[6] Yong C. Q. Y., Valiyaveettil S., Tang B. L. (2020). Toxicity of microplastics
and nanoplastics in Mammalian systems. Int.
J. Environ. Res. Public Health.

[7] Pinto
da Costa J., Reis V., Paço A., Costa M., Duarte A. C., Rocha-Santos T. (2019). Micro­(nano)­plastics
– Analytical challenges towards risk evaluation. TrAC, Trends Anal. Chem..

[8] Ivleva N. P. (2021). Chemical
Analysis of Microplastics and Nanoplastics: Challenges, Advanced Methods,
and Perspectives. Chem. Rev..

[9] Ter
Halle A., Jeanneau L., Martignac M., Jarde E., Pedrono B., Brach L., Gigault J. (2017). Nanoplastic
in the North Atlantic Subtropical Gyre. Environ.
Sci. Technol..

[10] Materić D., Kasper-Giebl A., Kau D., Anten M., Greilinger M., Ludewig E. (2020). Micro-and
Nanoplastics in Alpine Snow: A New Method
for Chemical Identification and (Semi)­Quantification in the Nanogram
Range. Environ. Sci. Technol..

[11] Materić D., Peacock M., Dean J., Futter M., Maximov T., Moldan F., Röckmann T., Holzinger R. (2022). Presence of
nanoplastics in rural and remote surface waters. Environ. Res. Lett..

[12] Materić D., Kjær H. A., Vallelonga P., Tison J. L., Röckmann T., Holzinger R. (2022). Nanoplastics measurements in Northern and Southern
polar ice. Environ. Res..

[13] Allen S., Allen D., Moss K., Le Roux G., Phoenix V. R., Sonke J. E. (2020). Examination of the ocean as a source for atmospheric
microplastics. PLoS One.

[14] Liu K., Wu T., Wang X., Song Z., Zong C., Wei N. (2019). Consistent Transport of Terrestrial Microplastics to
the Ocean through
Atmosphere. Environ. Sci. Technol..

[15] Wahl A., Le Juge C., Davranche M., El Hadri H., Grassl B., Reynaud S. (2021). Nanoplastic
occurrence in a soil amended with plastic
debris. Chemosphere.

[16] Davranche M., Lory C., Juge C. Le., Blancho F., Dia A., Grassl B. (2020). Nanoplastics on the coast exposed to the North Atlantic
Gyre: Evidence and traceability. NanoImpact.

[17] Tian L., Skoczynska E., van Putten R.-J., Leslie H. A., Gruter G.-J. M. (2023). Quantification
of polyethylene terephthalate micro- and nanoplastics in domestic
wastewater using a simple three-step method. Sci. Total Environ..

[18] Sendra M., Pereiro P., Yeste M. P., Mercado L., Figueras A., Novoa B. (2021). Size matters: Zebrafish
(Danio rerio) as a model to study toxicity
of nanoplastics from cells to the whole organism. Environ. Pollut..

[19] Davranche M., Lory C., Juge C. L., Blancho F., Dia A., Grassl B. (2020). Nanoplastics
on the coast exposed to the North Atlantic
Gyre: Evidence and traceability. NanoImpact.

[20] Boughbina-Portolés A., Campíns-Falcó P. (2024). Assessing
the size transformation
of nanoplastics in natural water matrices. Sci.
Total Environ..

[21] Mintenig S. M., Bäuerlein P. S., Koelmans A. A., Dekker S. C., Van Wezel A. P. (2018). Closing
the gap between small and smaller: towards a framework to analyse
nano- and microplastics in aqueous environmental samples. Environ. Sci. Nano.

[22] Gigault J., El Hadri H., Reynaud S., Deniau E., Grassl B. (2017). Asymmetrical
flow field flow fractionation methods to characterize submicron particles:
application to carbon-based aggregates and nanoplastics. Anal. Bioanal. Chem..

[23] Seeley M. E., Lynch J. M. (2023). Previous successes
and untapped potential of pyrolysis–GC/MS
for the analysis of plastic pollution. Anal.
Bioanal. Chem..

[24] Okoffo E. D., Thomas K. V. (2024). Mass quantification
of nanoplastics at wastewater treatment
plants by pyrolysis–gas chromatography–mass spectrometry. Water Res..

[25] Xu Y., Ou Q., Wang X., van der Hoek J. P., Liu G. (2024). Mass Concentration
and Removal Characteristics of Microplastics and Nanoplastics in a
Drinking Water Treatment Plant. ACS ES&T
Water.

[26] Junaid M., Liu S., Liao H., Yue Q., Wang J. (2024). Environmental nanoplastics
quantification by pyrolysis-gas chromatography–mass spectrometry
in the Pearl River, China: First insights into spatiotemporal distributions,
compositions, sources and risks. J. Hazard.
Mater..

[27] Huber M. J., Ivleva N. P., Booth A. M., Beer I., Bianchi I., Drexel R. (2023). Physicochemical
characterization and quantification
of nanoplastics: applicability, limitations and complementarity of
batch and fractionation methods. Anal. Bioanal.
Chem..

[28] ter
Laak T. L., Emke E., Benschop A., Nabben T., Béen F. (2022). Triangulating Amsterdam’s illicit stimulant
use trends by wastewater analysis and recreational drug use monitoring. Forensic Sci. Int..

[29] Water Quality — Sampling Part 10: Guidance on samplSng of wasteWwaterW International Organisation for Standardisation 2nd ed. 2020.

[30] Plastics Europe . Plastics-The Facts 2022, https://plasticseurope.org/knowledge-hub/plastics-the-facts-2022/. 2022.

[31] Wahlund K.-G. (2013). Flow field-flow
fractionation: Critical overview. J. Chromatogr
A.

[32] Parot J., Caputo F., Mehn D., Hackley V. A., Calzolai L. (2020). Physical characterization
of liposomal drug formulations using multi-detector asymmetrical-flow
field flow fractionation. J. Controlled Release.

[33] Lou F., Wang J., Sun C., Song J., Wang W., Pan Y. (2022). Influence of interaction on accuracy of quantification
of mixed microplastics using Py-GC/MS. J. Environ.
Chem. Eng..

[34] Alimi O. S., Farner J. M., Rowenczyk L., Petosa A. R., Claveau-Mallet D., Hernandez L. M. (2022). Mechanistic understanding of the aggregation
kinetics of nanoplastics in marine environments: Comparing synthetic
and natural water matrices. J. Hazard. Mater.
Adv..

[35] Andersson M., Wittgren B., Wahlund K. G. (2003). Accuracy
in multiangle light scattering
measurements for molar mass and radius estimations. Model calculations
and experiments. Anal. Chem..

[36] Giddings J.
C., Myers M. N. (1978). Steric
Field-Flow Fractionation: A New Method For Separating
I to 100 μm Particles. Sep. Sci. Technol..

[37] Galvão A., Aleixo M., De Pablo H., Lopes C., Raimundo J. (2020). Microplastics
in wastewater: microfiber emissions from common household laundry. Environ. Sci. Pollut. Res..

[38] Tian L., Skoczynska E., Siddhanti D., van Putten R. J., Leslie H. A., Gruter G. J. M. (2022). Quantification
of polyethylene terephthalate
microplastics and nanoplastics in sands, indoor dust and sludge using
a simplified in-matrix depolymerization method. Mar. Pollut. Bull..

[39] Makris K. F., Langeveld J., Clemens F. H. L. R. (2020). A review on the durability of PVC
sewer pipes: research vs. practice. Structure
and Infrastructure Engineering.

[40] Mintenig S. M., Kooi M., Erich M. W., Primpke S., Redondo-
Hasselerharm P. E., Dekker S. C. (2020). A systems approach to
understand microplastic occurrence and variability in Dutch riverine
surface waters. Water Res..

[41] Okoffo E. D., Rauert C., Thomas K. V. (2023). Mass quantification of microplastic
at wastewater treatment plants by pyrolysis-gas chromatography–mass
spectrometry. Sci. Total Environ..

[42] Koelmans A. A., Mohamed Nor N. H., Hermsen E., Kooi M., Mintenig S. M., De France J. (2019). Microplastics
in freshwaters and drinking water: Critical
review and assessment of data quality. Water
Res..

[43] Zwart N., Jonker W., Broek R. T., de Boer J., Somsen G., Kool J., Hamers T., Houtman C. J., Lamoree M. H. (2020). Identification
of mutagenic and endocrine disrupting compounds in surface water and
wastewater treatment plant effluents using high-resolution effect-directed
analysis. Water Res..

[44] Kaal E. R., Kurano M., Geiβler M., Janssen H.-G. (2008). Hyphenation of aqueous
liquid chromatography to pyrolysis-gas chromatography and mass spectrometry
for the comprehensive characterization of water-soluble polymers. J. Chromatogr A.

[45] Chojnacka A., Ghaffar A., Feilden A., Treacher K., Janssen H.-G., Schoenmakers P. (2011). Pyrolysis–gas
chromatography–mass spectrometry
for studying N-vinyl-2-pyrrolidone-co-vinyl acetate copolymers and
their dissolution behaviour. Anal. Chim. Acta.

[46] Ventouri I. K., Chang W., Meier F., Drexel R., Somsen G. W., Schoenmakers P. J. (2023). Characterizing Non-covalent Protein Complexes
Using Asymmetrical Flow Field-Flow Fractionation On-Line Coupled to
Native Mass Spectrometry. Anal. Chem..

[47] Meili-Borovinskaya O., Meier F., Drexel R., Baalousha M., Flamigni L., Hegetschweiler A. (2021). Analysis of complex
particle mixtures by asymmetrical flow field-flow fractionation coupled
to inductively coupled plasma time-of-flight mass spectrometry. J. Chromatogr A.

[48] Sørensen L., Gerace M. H., Booth A. M. (2024). Small micro-
and nanoplastic test
and reference materials for research: Current status and future needs. Cambridge Prisms: Plastics.

