# Supplementary material for: Integrating AF4 and Py-GC-MS for Combined Size-Resolved Polymer-Compositional Analysis of Nanoplastics with Application to Wastewater
Source: Anal Chem. 2025 Jul 11;97(28):15216–24. doi: 10.1021/acs.analchem.5c01766 (PMC12291041; doi:10.1021/acs.analchem.5c01766)
Supplement: Supplementary file 1 [file ac5c01766_si_001.pdf]

Supporting Information

Integrating AF4 and Py-GC-MS for combined size-resolved polymer-compositional analysis of nanoplastics with application to wastewater

Maria Hayder[1*], Cloé Veclin[1], Aislinn Ahern[1], Aleksandra Chojnacka[1], Erwin Roex[2], Florian Meier[3], Gert-Jan Gruter[1,4], Annemarie van Wezel[5], Alina Astefanei[1]

[1]Van't Hoff Institute for Molecular Sciences, University of Amsterdam, Science Park 904, 1098XH, Amsterdam, Netherlands

[2]National Institute for Public Health and the Environment (RIVM), 3720BA, Bilthoven, Netherlands

[3]Postnova Analytics GmbH, Rankinestraße 1, 86899 Landsberg, Germany

[4]Avantium Support BV, Zekeringstraat 29, 1014BV, Amsterdam, Netherlands

[5]Institute for Biodiversity and Ecosystem Dynamics, University of Amsterdam, Science Park 904, 1098XH, Amsterdam, Netherlands

*Corresponding author: Maria Hayder, m.w.hayder@uva.nl

Contents

Section S1. Chemicals 2

Section S2. AF4-UV-MALS instrumentation 2

Figure S1. A) AF4 separation channel. B) In- and outflows of the AF4 channel 3

Section S3. LVI-AF4 carrier liquid optimization 3

Table S1. DLS results of PSL50 agglomeration behavior in different salt solutions. All salt solutions had ionic strength of 0.5 mM. 4

Section S4. Flow rate program and recovery in AF4 measurements 4

Section S5. Py-GC-MS instrumentation 5

Table S2. PTV inlet program for the analysis of liquid samples 5

Table S3. GC oven program 5

Table S4. MS experimental parameters 6

Table S5. Conditions of polymer dissolution for Py-GC/MS experiments 6

Table S6. Pyrolysis products of target polymers and their analysis. *m/z* in bold were used for quantification. LOD=a+3.3·b, LOQ=a+10·b, where a is an average signal in 26 blank measurements and b is the standard deviation. 6

Section S6. Filtration and freeze-drying validation 6

Figure S2. Separation and size measurements of PSC50 (peak 1) and PSC200 (peak 2) mixture using SVI (black trace) and LVI (injection time 90 mins, orange trace). Injection times are subtracted from the fractograms. 7

Table S7. Pyrolysis products of target polymers in literature 7

Figure S3. Chromatograms of solid injections of polyolefins. A) *m/z* 69 B) *m/z* 97. Pink PE, blue PP, black PE+PP 9

Figure S4. Py-GC/MS calibration curves of (a) PS (5.5-550 ng); (b) PVC (5.5-275 ng); and PET (27.5-550 ng); and (c) PE (27.5-550 ng) and PP (27.5-550 ng). 11

Figure S5. MALS 90° traces of LVI-AF4 blanks, filtered and unfiltered. Compared to samples, all signals are negligible 11

Section S7. Py-GC-MS of PE and PP 11

References 12

Section S1. Chemicals

Ammonium carbonate (purity >99%), was purchased from Merck (Darmstadt, Germany). Polyvinylchloride (PVC), polypropylene (PP), low-density polyethylene (PE) and 1,2,4-trichlorobenzene (TCB) (≥98%) were purchased from Merck. Polyethylene terephthalate (PET) was donated by Avantium BV, Amsterdam, The Netherlands. Polystyrene (PS, Mp 2970) was purchased from Polymer Laboratories Ltd (Church Stretton, UK). Tetrahydrofuran (THF) (≥99.8%) was purchased from Biosolve BV (Valkenswaard, The Netherlands). P-xylene (99%) was purchased from Alfa Aesar (Haverhill, MA, USA). Hexafluoroisopropanol (HFIP) was recovered in-house. Non-modified polystyrene particles (50 nm diameter) were purchased from Polysciences Inc. (Warrington, PA, USA).

Section S2. AF4-UV-MALS instrumentation

AF4-UV-MALS measurements were performed using an AF4-UV-MALS system (AF2000 MultiFlow FFF system, Postnova Analytics, Landsberg am Lech, Germany) with an SPD-20A UV/Vis absorbance detector operated at 280 nm and 254 nm (PN3212;

Shimadzu, Kyoto, Japan) and a 21-angle MALS detector (PN3621). Data was acquired by the AF2000 control software version 2.1.0.1 (Postnova Analytics). Radii of gyration (r_g) were derived using the Sphere model for PSC and Berry model (degree 3) for wastewater samples. During r_g calculations, some angles were excluded due to their low signal-to-noise ratio. For PSC, angles between 28° and 156° were included, while for the wastewater samples, angles between 20° and 156° were included. Fractions were collected using a Fraction Collector III (Waters, Milford, MA, USA).

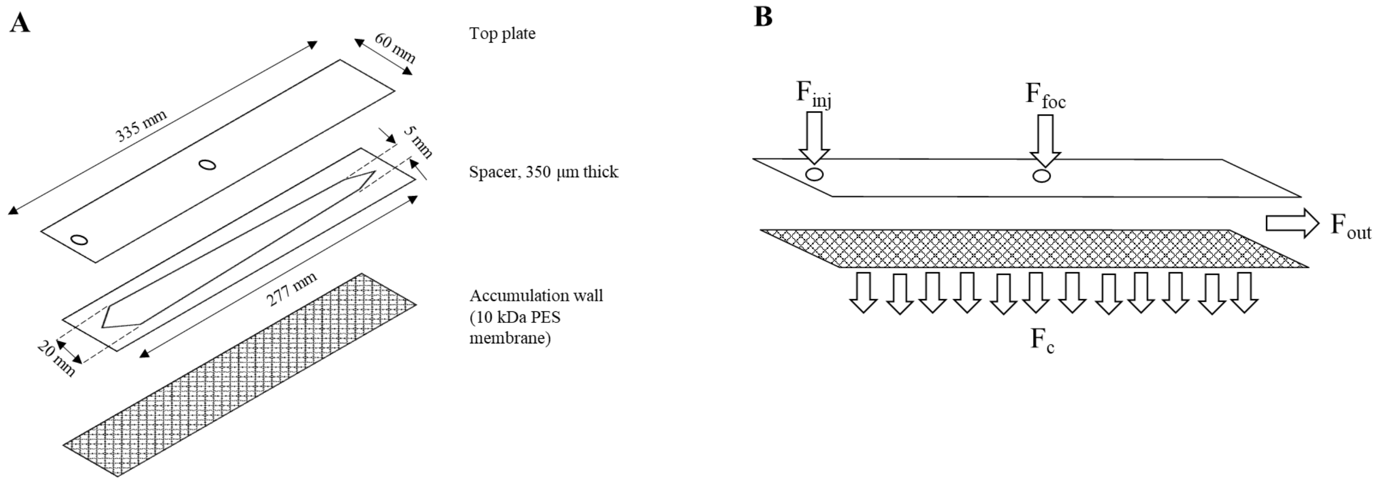

Figure S1. A) AF4 separation channel. B) In- and outflows of the AF4 channel

Section S3. LVI-AF4 carrier liquid optimization

The AF4 method was developed on PSC standard particles. To prevent interference with subsequent Py-GC-MS measurements and instrumental disruptions, surfactants and non-volatile salts, commonly mentioned as AF4 carrier liquid for PS particles,[1–3] were excluded. To address the risk of particle agglomeration during LVI, various volatile salts (ammonium formate, sodium carbonate, ammonium nitrate, potassium nitrate, ammonium carbonate, Merck, Darmstadt, Germany) were evaluated. Each carrier liquid was prepared by dissolving the respective salt in ultrapure water (resistivity 18.2 MΩ; Sartorius Arium 611UV; Sartorius, Göttingen, Germany) to a final ionic strength of 0.5 mM. PSL50 particles were diluted to a final

concentration of 323 µg/mL in each salt solution. The hydrodynamic radius in each suspension was measured by DLS immediately after preparation, and again after 1 after 1 h in 11-21 replicates, depending on the measurement quality. Statistical significance of size differences was evaluated using a Student t-test.

DLS measurements were performed at 25°C using a quartz cuvette of 1 µL on a DynaPro NanoStar system (Wyatt Technology, Dernbach, Germany). DLS data was acquired by Dynamics software version 7.4.0.72 (Wyatt Technology). To derive the mean values of the measured hydrodynamic radii, regularization analysis with intensity-weighting was used.

Table S1. DLS results of PSL50 agglomeration behavior in different salt solutions. All salt solutions had ionic strength of 0.5 mM.

Salt	p value
NH_4HCO_2	0.000142 (size increased by 3.9 nm)
Na_2CO_3	0.041651 (size increased by 4.9 nm)
NH_4NO_3	0.048181 (size increased by 2.3 nm)
KNO_3	0.61306
$(NH_4)_2CO_3$	0.20115
$NaNO_3$	0.712613

Sodium nitrate, potassium nitrate and ammonium carbonate did not cause a significant size increase of the particles.

Section S4. Flow rate program and recovery in AF4 measurements.

During elution, the crossflow rate was exponentially decreased (exponent=0.2) from 1 mL/min to 0.1 mL/min over 25 mins. Then, it was kept constant for 15 mins at 0.1 mL/min and for 10 mins at 0 mL/min. Detector flow was kept at 0.5 mL/min.

For recovery experiments, 1 μL of the stock suspension was injected as SVI with and without crossflow applied. Subsequently, the corresponding dilutions of the same batch of the stock suspensions were injected as LVI and measured. The respective peaks of the UV signal were compared to calculate the AF4 recovery.

Section S5. Py-GC-MS instrumentation

Py-GC-MS measurements were performed on a Shimadzu GCMS-QP2010 Plus system (Kyoto, Japan) with an Optic-4 programmed-temperature vaporization (PTV) injector (ATAS GL, Veldhoven, The Netherlands) and a Focus XYZ autosampler (ATAS). The PTV injector acts as the pyrolysis chamber and is embedded in the GC oven, thus preventing appearance of a "cold spot" on the pyrolyzer-GC interface, which minimizes losses of high-molecular weight compounds. In the liquid injection the sample is distributed in a glass tube (liner) space where the pyrolysis temperature is well defined. The Py-GC-MS measurements performed with the PTV injector are described as repeatable.[4] The limitations of PTV are: i) heating speed lower than in conventional pyrolyzers; ii) upper limit of injection volume and iii) temperature vulnerability of glass liners, which cannot withstand temperatures higher than 600˚C.

A DB-17MS column (30 m long, ID 0.250 mm, 0.25 μm film thickness) (Agilent Technologies, Santa Clara, CA, USA) with helium as a carrier gas at the flow rate 1.5 mL/min was used.

Table S2. PTV inlet program for the analysis of liquid samples

Time (s)	Inlet temperature (˚C)	Split flow (ml/min)
0	50	200
20	50-150	200
110	150	200
40	150	10
9	150-550	10

Table S3. GC oven program

Start temperature (˚C)	Final temperature (˚C)	Time (min)
50	50	4
50	320	27

320	320	5

Table S4. MS experimental parameters

Parameter	Value
Ion source temperature (°C)	200
Interface temperature (°C)	280
Acquisition interval (s)	0.05
Scan speed (-)	10000
Start time (min)	6.1

Table S5. Conditions of polymer dissolution for Py-GC/MS experiments

Polymer	Solvent	Dissolving conditions
PS	THF	-
PVC	THF	70°C, 300 rpm
PET	Hexafluoroisopropanol (HFIP)	70°C, 300 rpm
PE	1:1mixture of p-xylene and 1,2,4-trichlorobenzene	70-150°C, 550 rpm
PP		

Table S6. Pyrolysis products of target polymers and their analysis. *m/z* in bold were used for quantification. LOD=a+3.3·b, LOQ=a+10·b, where a is an average signal in 26 blank measurements and b is the standard deviation.

Polymer	Pyrolysis products	*m/z*	tr (min)	LOD and LOQ (ng)
**PS**	styrene<br>styrene dimer<br>**styrene trimer**	78, 104<br>91, 104, 130, 208<br>**91**, 117, 207, 312	26.5	LOD 0.22<br>LOQ 0.64
**PVC**	**naphthalene**<br>styrene<br>indene	**128**<br>78, 104<br>115, 116, 142	12.5	LOD 21.1<br>LOQ 54.0
**PET**	**acetophenone**<br>benzoic acid	77, **105**<br>105, 122	11.4	LOD 27.5<br>LOQ 55.0
**polyolefins**	**α-alkenes**	69, 83, **97**, 111 (PE)	15.0	LOD 86.0<br>LOQ 180
		**69**, 83, 97, 111 (PP)	13.8	LOD 38.8<br>LOQ 88.1

Section S6. Filtration and freeze-drying validation

The influence of filtration on NPs was investigated using PSC50 and PSC200 suspensions. Three filtered replicates and three unfiltered ones were measured with LVI-AF4-UV-MALS. Filtration recovery and potential of altering particle size distributions were assessed by

comparing the UV peak areas and the radii of gyration (r_g), respectively. As control, the filtration was tested by performing the same procedure with carrier liquid only.

Freeze-drying recovery was measured by adding 50 µL of undiluted PSC50 or PSC200 suspension to pre-weighed vials, subsequent freeze-drying and reweighing in triplicates. Small plastic vials were used here to accurately measure slight mass differences.

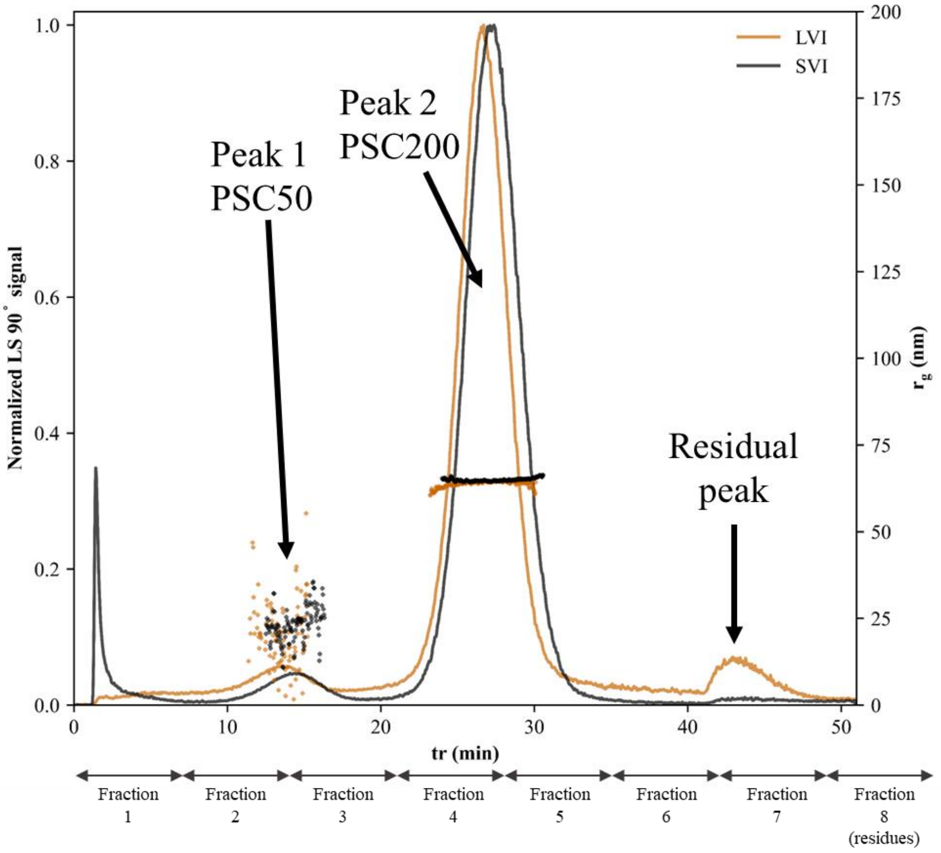

Figure S2. Separation and size measurements of PSC50 (peak 1) and PSC200 (peak 2) mixture using SVI (black trace) and LVI (injection time 90 mins, orange trace). Injection times are subtracted from the fractograms.

Table S7. Pyrolysis products of target polymers in literature

polymer	decomposition products	m/z	reference
**PS**	Styrene<br>Styrene dimer<br>Styrene trimer	104,78,51<br>208; 208, 130, 104,91<br>312; 207, 194, 117, 91	3,5–9<br>5–8<br>6–9
**PVC**	Benzene<br>Chlorobenzene<br>*Indicative ion*	78; 78,52<br>112; 112,77<br>*78*	5<br>6,7<br>10

	1-methylnaphthalene	142	11,12
	2-methylnaphthalene	142	11
	Styrene	104	13
	Mesitylene	120	13
	Indene	116	13
	Naphthalene	128	13
	Biphenyl	154	13
	Acenaphthalene	154	13
	Diethyl phthalene	222	13
	Anthracene	178	13
	Pyrene	202	13
**PET**	Dimethyl terephthalate	194; 194,163	6,7
	Benzene	78,52	5
	Acetophenone	105,77,51	5
	Vinyl benzoate	148,105,77,52,51	5,7,8,12
	Benzoic acid	122,105,77,51	5,8,12
	Divinyl terephthalate	175,104	5
	Ethyl benzoate	150,122,105,77	7,8
	1,1-biphenyl	154,105,76	7,8,12
	(TMAH) dimethyltherephthalate	163,194	12
	(TMAH) ethan-1,2-diyldibenzoate	227,105	12
	(TMAH) 2-(benzoyloxy)ethylvinyl terephthalate	297	12
**PP**	2,4-dimethylhept-1-ene	126; 126,83,70, 43	5–9,12
	2,4,6,8-tetramethyl-1-undecene	210;154,125,100,111,83,69	6–9
	Indicative ion	70	10
	Oligomers with higher chain length (2,4,6,8-tetramethyl-1-undecene)	69	10
	2,4,6-trimethylnon-1-ene	125,111,69,43	8,9
**PE**	Alkanes	282; 99,85	6,7
	α-alkanes	280; 97,83	6,7
	αω-alkanes	278; 95,55,82	6,7
	1-nonene	83,97	5
	1-decene	83,97	5
	1-unodecene	83,97	5
	1-dodecene	83,97	5
	1-tridecene	83,97	5
	1-tetradecene	83,97	5
	1-pentadecene	83,97	5
	1-hexadecene	83,97	5
	Indicative ion	*85,82*	10
	1,12-tridecadiene	95,67,81,55	8
	1.13-tetreadecadiene	109,95,81	8
	1,14-pentadecadiene	109,95,81,55	8
	1,15-hexadecadiene	96,81,69,55	8

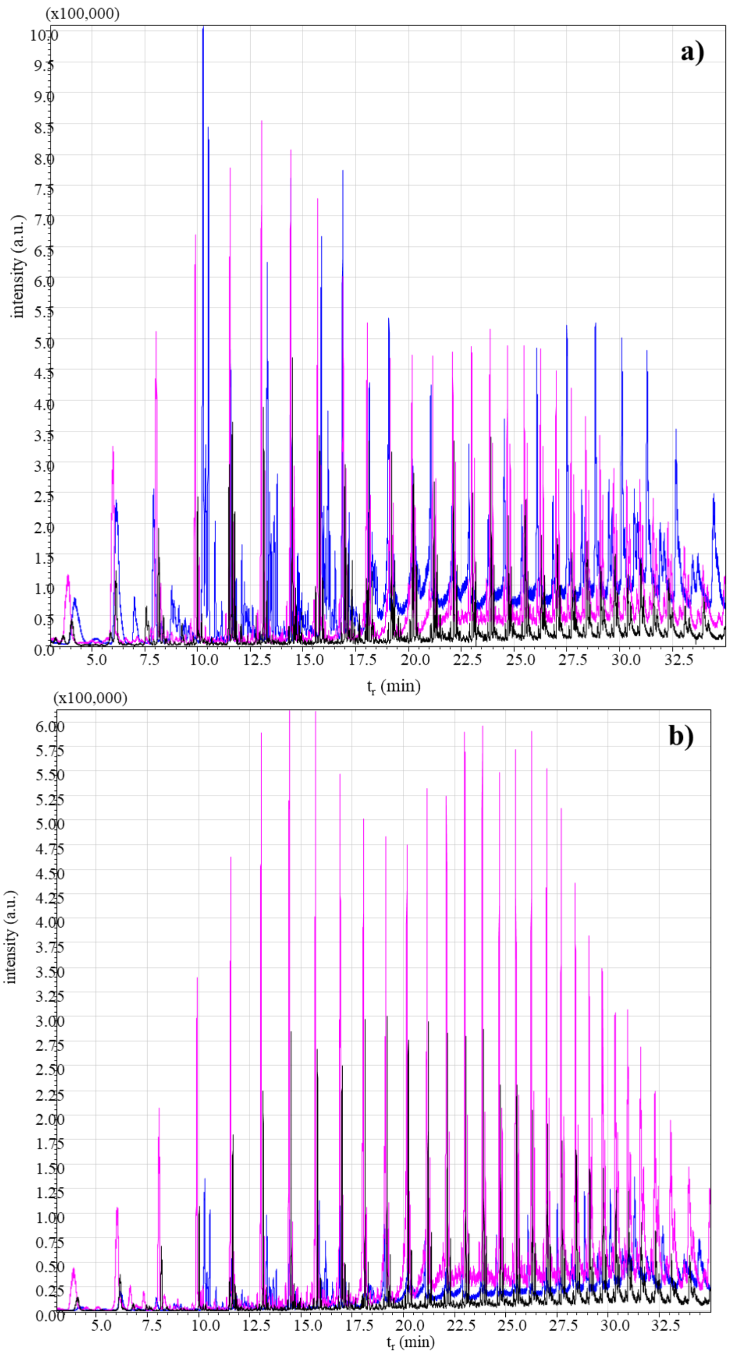

Figure S3. Chromatograms of solid injections of polyolefins. A) *m/z* 69 B) *m/z* 97. Pink PE, blue PP, black PE+PP

a

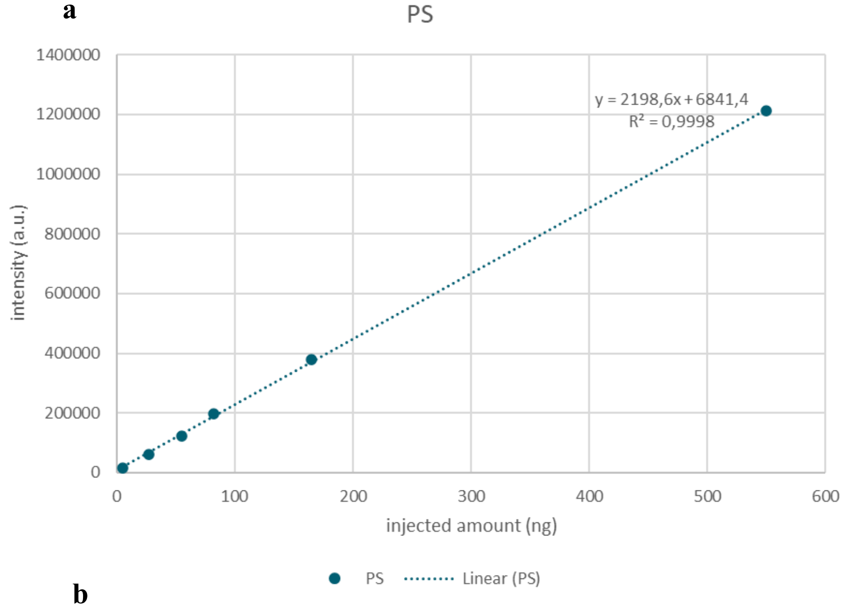
PS
1400000
1200000
1000000
800000
600000
400000
200000
0
0
100
200
300
400
500
600
intensity (a.u.)
injected amount (ng)
y = 2198,6x + 6841,4
R² = 0,9998
PS
Linear (PS)

b

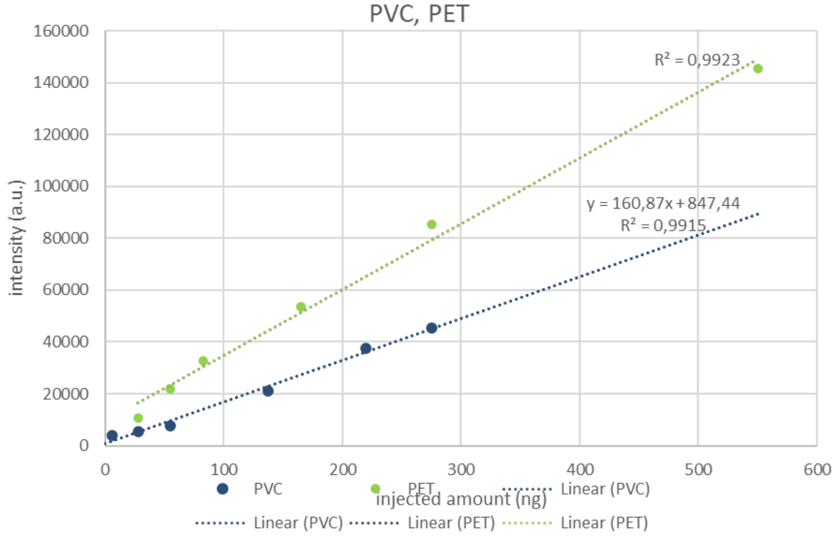
PVC, PET
160000
140000
120000
100000
80000
60000
40000
20000
0
0
100
200
300
400
500
600
intensity (a.u.)
injected amount (ng)
R² = 0,9923
y = 160,87x + 847,44
R² = 0,9915
PVC
PET
Linear (PVC)
Linear (PVC)
Linear (PET)
Linear (PET)

c

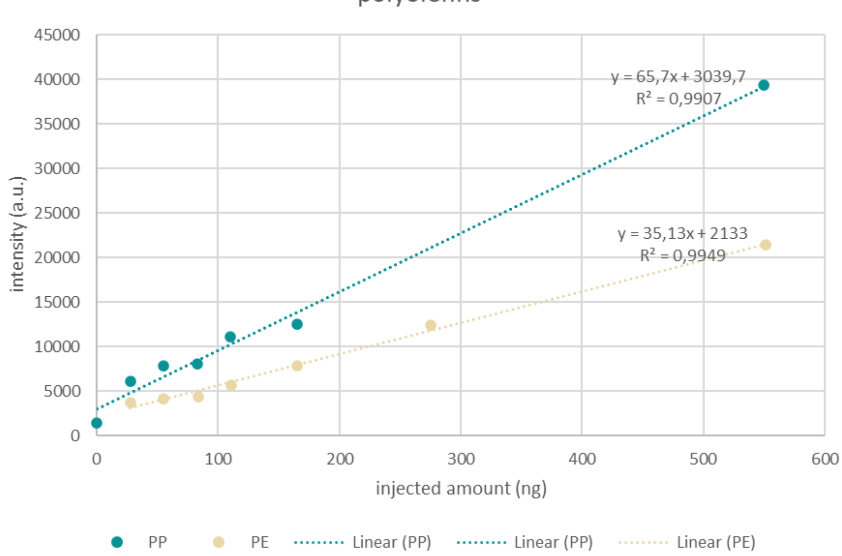
polyolefins
45000
40000
35000
30000
25000
20000
15000
10000
5000
0
0
100
200
300
400
500
600
intensity (a.u.)
injected amount (ng)
y = 65,7x + 3039,7
R² = 0,9907
y = 35,13x + 2133
R² = 0,9949
PP
PE
Linear (PP)
Linear (PP)
Linear (PE)

Figure S4. Py-GC/MS calibration curves of (a) PS (5.5-550 ng); (b) PVC (5.5-275 ng); and PET (27.5-550 ng); and (c) PE (27.5-550 ng) and PP (27.5-550 ng).

The amounts injected were (in ng): PS: 5.5, 27.5, 82.5, 165, 275, 550; PVC: 5.5, 27.5, 55, 137.5, 220, 275; PET: 27.5, 55, 82.5, 110, 165, 275, 550; PE: 27.5, 55, 83.6, 110.6, 165, 275.6, 551.1; PP: 0, 27.5, 55, 82.5, 110, 165, 550.

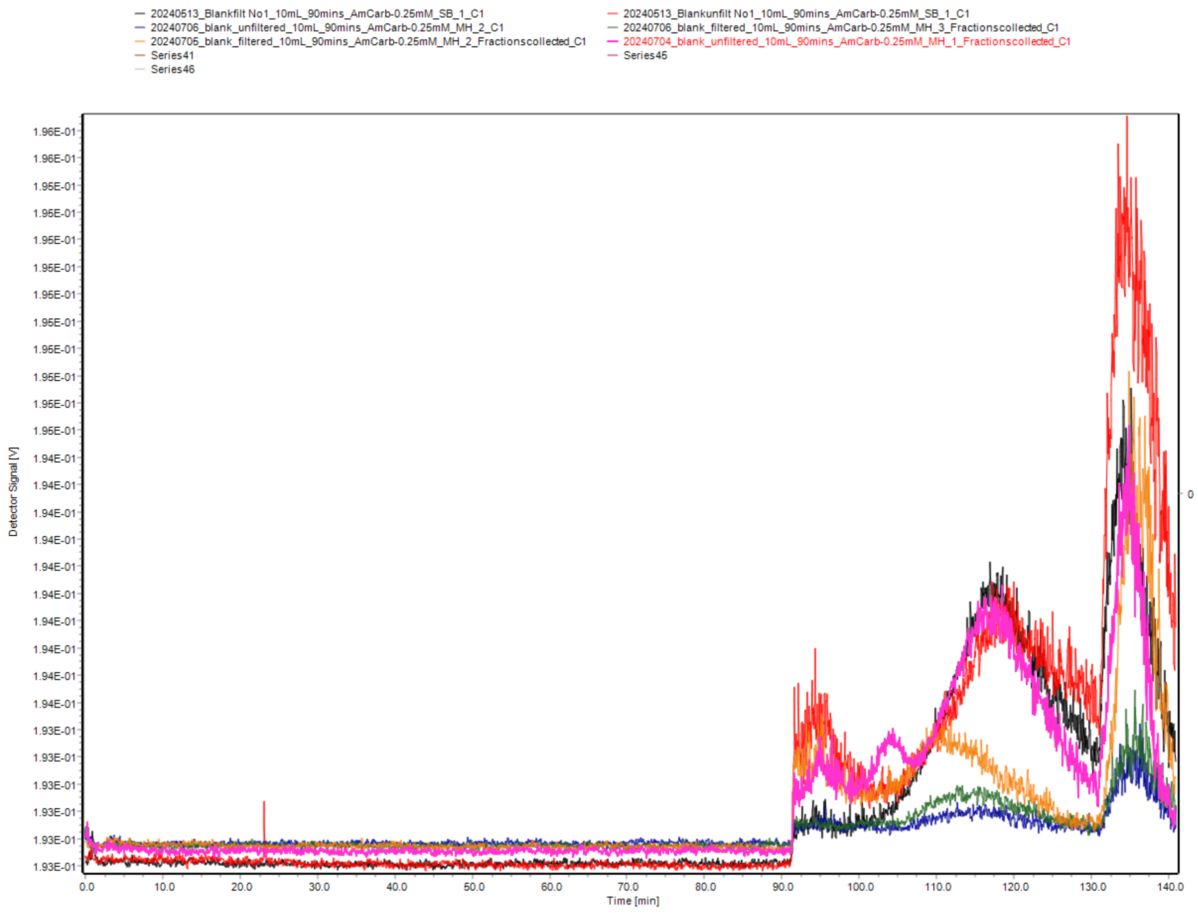

Figure S5. MALS 90° traces of LVI-AF4 blanks, filtered and unfiltered. Compared to samples, all signals are negligible

Section S7. Py-GC-MS of PE and PP

Due to PE and PP being among the most massively produced polymers,[14] their reliable analysis is of high importance in micro- and nanoplastics research. However, due to the similar molecular structure of these two polymer families, they yield similar chromatograms and the respective mass spectra (see Figure S3), creating a serious hurdle in reliable Py-GC-MS of polyolefins. As shown in Figure S3, the signals obtained from the measurement of both PE and

PP lie very close to each other. GC×GC-MS might be a way to improve the separation and thus bring more confidence about the chemistry of the analytes.

References

1 Luo XL *et al.* An effective solution to simultaneously analyze size, mass and number concentration of polydisperse nanoplastics in a biological matrix: asymmetrical flow field fractionation coupled with a diode array detector and multiangle light scattering. *RSC Adv* 2021; **11**. doi:10.1039/d1ra00450f.

2 Gigault J *et al.* Asymmetrical flow field flow fractionation methods to characterize submicron particles: application to carbon-based aggregates and nanoplastics. *Anal Bioanal Chem* 2017; **409**. doi:10.1007/s00216-017-0629-7.

3 Mintenig SM *et al.* Closing the gap between small and smaller: towards a framework to analyse nano- and microplastics in aqueous environmental samples. *Environ Sci Nano* 2018; **5**. doi:10.1039/c8en00186c.

4 Hermabessiere L *et al.* Optimization, performance, and application of a pyrolysis-GC/MS method for the identification of microplastics. *Anal Bioanal Chem* 2018; **410**: 6663–6676.

5 Fischer M, Scholz-Böttcher BM. Simultaneous Trace Identification and Quantification of Common Types of Microplastics in Environmental Samples by Pyrolysis-Gas Chromatography–Mass Spectrometry. *Environ Sci Technol* 2017; **51**: 5052–5060.

6 Primpke S *et al.* Critical Assessment of Analytical Methods for the Harmonized and Cost-Efficient Analysis of Microplastics. *Appl Spectrosc* 2020; **74**: 1012–1047.

7 Dümichen E *et al.* Fast identification of microplastics in complex environmental samples by a thermal degradation method. *Chemosphere* 2017; **174**: 572–584.

8 Shin T *et al.* Pyrograms and Thermograms of 163 High Polymers, and MS Data of the Major Pyrolyzates. In: *Pyrolysis ‒ GC/MS Data Book of Synthetic Polymers*. Elsevier, 2011, pp 7–335.

9 Käppler A *et al.* Comparison of μ-ATR-FTIR spectroscopy and py-GCMS as identification tools for microplastic particles and fibers isolated from river sediments. *Anal Bioanal Chem* 2018; **410**: 5313–5327.

10 Okoffo ED, Thomas K V. Quantitative analysis of nanoplastics in environmental and potable waters by pyrolysis-gas chromatography–mass spectrometry. *J Hazard Mater* 2024; **464**: 133013.

11 La Nasa J *et al.* A review on challenges and developments of analytical pyrolysis and other thermoanalytical techniques for the quali-quantitative determination of microplastics. *J Anal Appl Pyrolysis* 2020; **149**: 104841.

12 Peters CA *et al.* Pyr-GC/MS analysis of microplastics extracted from the stomach content of benthivore fish from the Texas Gulf Coast. *Mar Pollut Bull* 2018; **137**: 91–95.
